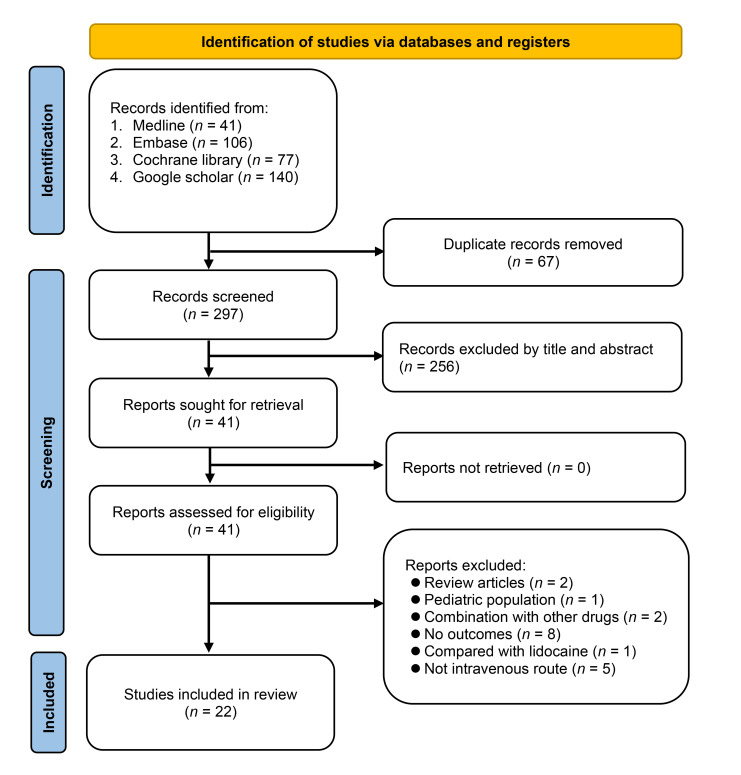# Impact of intravenous dexmedetomidine on postoperative gastrointestinal function recovery: an updated meta-analysis

**DOI:** 10.1097/JS9.0000000000000988

**Published:** 2023-12-12

**Authors:** Yi-Chen Lai, Wei-Ting Wang, Kuo-Chuan Hung, Jen-Yin Chen, Jheng-Yan Wu, Ying-Jen Chang, Chien-Ming Lin, I-Wen Chen

**Affiliations:** a Department of Anesthesiology, Chi Mei Medical Center, Tainan city, Taiwan; b Department of Anesthesiology, E-Da Hospital, I-Shou University, Kaohsiung, Taiwan; c School of Medicine, College of Medicine, National Sun Yat-sen University, Kaohsiung, Taiwan; d Department of Nutrition, Chi Mei Medical Center, Tainan City, Taiwan; e Department of Anesthesiology, Chi Mei Medical Center, Liouying, Tainan city, Taiwan

**Keywords:** dexmedetomidine, gastrointestinal function, meta-analysis, postoperative ileus, surgery

## Abstract

**Background:**

Postoperative ileus (POI) is a complication that may occur after abdominal or nonabdominal surgery. Intravenous dexmedetomidine (Dex) has been reported to accelerate postoperative gastrointestinal function recovery; however, updated evidence is required to confirm its robustness.

**Methods:**

To identify randomized controlled trials examining the effects of perioperative intravenous Dex on gastrointestinal function recovery in patients undergoing noncardiac surgery, databases including MEDLINE, EMBASE, Google Scholar, and Cochrane Library were searched on August 2023. The primary outcome was time to first flatus. Secondary outcomes included time to oral intake and defecation as well as postoperative pain scores, postoperative nausea/vomiting (PONV), risk of hemodynamic instability, and length of hospital stay (LOS). To confirm its robustness, subgroup analyses and trial sequential analysis were performed.

**Results:**

The meta-analysis of 22 randomized controlled trials with 2566 patients showed that Dex significantly reduced the time to flatus [mean difference (MD):−7.19 h, *P*<0.00001), time to oral intake (MD: −6.44 h, *P*=0.001), time to defecation (MD:−13.84 h, *P*=0.008), LOS (MD:−1.08 days, *P*<0.0001), and PONV risk (risk ratio: 0.61, *P*<0.00001) without differences in hemodynamic stability and pain severity compared with the control group. Trial sequential analysis supported sufficient evidence favoring Dex for accelerating bowel function. Subgroup analyses confirmed the positive impact of Dex on the time to flatus across different surgical categories and sexes. However, this benefit has not been observed in studies conducted in regions outside China.

**Conclusions:**

Perioperative intravenous Dex may enhance postoperative gastrointestinal function recovery and reduce LOS, thereby validating its use in patients for whom postoperative ileus is a significant concern.

## Introduction

Highlights

- The efficacy of dexmedetomidine (Dex) for postoperative ileus was investigated.
- Dex was linked to shorter times to first flatus, oral feeding, and defecation.
- Hemodynamic instability and postoperative pain severity were similar with or without Dex use.
- Dex was associated with lower risk of nausea/vomiting and shorter hospital stay.
- Trial sequential analysis favored Dex in promoting bowel function.

Postoperative ileus (POI) is characterized by a prolonged delay in the return of normal gastrointestinal motility and tolerance of an oral diet. While a certain degree of impaired gastrointestinal function in the first 1–2 days after surgery is an expected physiologic response, POI refers to the pathological persistence of absent or severely reduced bowel sounds, inability to tolerate oral intake, nausea/vomiting, and abdominal distension lasting greater than 3–5 days^1,2^. Although POI is predominantly observed following gastrointestinal surgery^3^, it can also occur in diverse surgical types, including lumbar fusion surgery, owing to extended immobilization. POI not only prolongs hospital stays and diminishes patient satisfaction but also escalates healthcare costs and intensifies the demand on medical resources^4,5^. A recent meta-analysis showed that patients with POI incur an additional €8233 in costs, translating to a 66.3% increase in total hospital expenses^6^. Sympathetic reflex activation, inflammation triggered by surgical trauma and bowel manipulation, perioperative opioid use, and prolonged immobility are potential mechanisms and factors contributing to POI^7,8^. The incidence of POI is ~10–20%^9–11^, underscoring the significance of implementing strategies to prevent its occurrence.

Dexmedetomidine (Dex), a potent and highly selective alpha-2 adrenoreceptor agonist, is frequently employed as an anesthetic adjunct in surgical procedures^12^. By reducing the surgical stress response through its central sympatholytic and anti-inflammatory effects, Dex offers distinct organ protection^12^. Furthermore, its opioid-sparing effect reduces the need for perioperative analgesics^13,14^. Two recent meta-analyses^15,16^ reported a positive impact of perioperative Dex use on postoperative gastrointestinal function by shortening the time to flatus. However, the high heterogeneity (e.g. *I* ^2^=87%^15^ and *I* ^2^=95%^16^) and relatively small sample sizes (e.g. 362^15^ and 1090^16^ participants) in these previous analyses made their conclusions less robust. Furthermore, sources of heterogeneity have not been thoroughly evaluated. In light of the growing research interest in this topic in recent years, an updated and more comprehensive meta-analysis is warranted to provide stronger evidence regarding the effects of Dex on postoperative bowel function.

This meta-analysis aimed to investigate the impact of perioperative Dex use on postoperative bowel function recovery by examining the time to first flatus in adult patients undergoing noncardiac surgery. The secondary objectives were to evaluate the impact of Dex on the time to oral intake, time to defecation, length of hospital stay (LOS), postoperative pain scores, and its potential correlation with the risk of postoperative nausea and vomiting (PONV) and hemodynamic instability (e.g. hypotension or bradycardia).

## Method

The protocol for the current meta-analysis was registered in PROSPERO. This study adhered to the Preferred Reporting Items for Systematic Reviews and Meta-Analyses Statement (PRISMA)^17^ (Supplemental Digital Content 1, http://links.lww.com/JS9/B564) (Supplemental Digital Content 2, http://links.lww.com/JS9/B565) and AMSTAR (Assessing the methodological quality of systematic reviews)^18^ guidelines when reporting its findings (Supplemental Digital Content 3, http://links.lww.com/JS9/B566).

### Literature search

Through a comprehensive literature review, we examined the potential benefits of intravenous Dex on gastrointestinal function recovery. We searched several databases, including EMBASE, MEDLINE, Cochrane Library, and Google Scholar, from their inception to 20 August 2023. Using a combination of keywords and MeSH terms such as (‘dexmedetomidine’ or ‘GI function recovery’ or ‘gastrointestinal motility’ or ‘postoperative ileus’), we identified studies addressing the association between intravenous Dex and gastrointestinal function recovery. Notably, during the search process, no restrictions were imposed on language, sex distribution, publication year, or sample size. In addition to this comprehensive exploration, we also scrutinized the reference lists of pertinent systematic reviews and included reports. The search methodology and terms used are presented in Supplemental Table 1 (Supplemental Digital Content 4, http://links.lww.com/JS9/B567).

### Study selection and inclusion criteria

Randomized controlled trials (RCTs) were considered eligible if the following criteria were met.

1. Population: adult patients undergoing general anesthesia for noncardiac surgery, without the need for ICU admission postoperatively.
2. Intervention: intravenous Dex was administered perioperatively, including its use following surgery (e.g. Dex used as a supplement for patient-controlled analgesia).
3. Comparator: the use of standard care, placebos (e.g. normal saline), or opioids was considered eligible for the control group.
4. Outcomes: studies reporting time to flatus.

The excluded studies were (1) nonhuman studies, case reports, conference proceedings, letters, and reviews; (2) studies that used lidocaine or ketamine as comparators; and (3) studies in which Dex was not intravenously administered. For instance, it has been used as a supplement for local infiltration, nerve block, or epidural analgesia.

Two authors independently reviewed the titles and abstracts of retrieved articles. A discussion was held to settle all conflicts. A full-text review was performed on the articles that passed the abstract and title screenings, and a final analysis was performed following author agreement.

### Data extraction

Two independent authors collected the following information from each study: age and sex of participants, surgical type, number of participants, surgical time, Dex dosage, timing of Dex delivery (such as intraoperative or postoperative); time to flatus; time to defecation; postoperative pain severity; LOS; time to first oral intake; incidences of hypotension, bradycardia, or PONV; and country in which the study was conducted. In a triple-arm study design, where normal saline/opioids or lidocaine/ketamine are employed as control groups, data extraction will solely encompass the normal saline/opioids group. If deemed necessary, the researchers of the included studies were asked to gather any absent details or seek further elucidation of the data.

### Quality assessment

In accordance with the framework of risk of bias (ROB) 2.0, two independent authors performed a comprehensive assessment of the risk of bias for the studies included in this meta-analysis. Five distinct domains of bias, including randomization, intervention deviations, missing data, outcome measurement, and result reporting, were meticulously scrutinized. A study was classified as a ‘low risk’ study only if it demonstrated a low risk of bias across all five domains. In cases where a trial raised ‘some concerns’ in at least one domain, it was categorized as having ‘some concerns’. If a study exhibited a high risk of bias in one or more domains, it was designated as having an overall ‘high risk’ of bias. We assessed each study’s bias risk by applying the ROB 2.0’s criteria and signaling questions.

### Outcomes and definitions

The primary outcome was time to flatus, whereas the secondary outcomes included time to defecation, time to first oral intake, postoperative pain severity at 12–24 h, LOS, and risks of hypotension, bradycardia, or PONV. We selected time to flatus as the primary outcome because it is reportedly the most commonly used measurement to describe bowel function recovery^19^. Subgroup analyses of the primary outcome were performed on the basis of the type of surgical approach (i.e. laparoscopy vs. nonlaparoscopy; abdominal vs. nonabdominal surgery), sex (e.g. female vs. mixed-sex), and country (China vs. non-China).

### Data synthesis

The correlation between Dex and postoperative recovery was quantified as an effect size, presented as the mean difference (MDs) with a 95% CI for continuous outcomes, or as a risk ratio (RR) for a dichotomous variable. The Mantel–Haenszel random-effects model was used for the analysis of outcomes, with careful consideration provided to the heterogeneity present in clinical-related and population-related variables. The level of heterogeneity evaluated using *I* ^2^ statistics was deemed substantial when it exceeded 50%. To ascertain the impact of each study’s findings, a leave-one-out sensitivity analysis was performed for the outcomes with *I* ^2^ >50%. To identify potential publication bias, a funnel plot centered on a particular outcome outlined in 10 or more datasets was examined for symmetry. Two-tailed tests were employed for all comparisons, considering a *P*-value of <0.05 as indicative of statistical significance. Data were synthesized using the Cochrane Review Manager (RevMan 5.3; Copenhagen, The Nordic Cochrane Center).

Participant numbers were divided to prevent redundancy in the assessment of sample size within studies featuring multiple intervention arms. In cases where a study consisted of two intervention groups and one control group, the number of patients within the control group was equally divided for comparison with the corresponding intervention groups. For continuous outcomes, altering the means and SD was not required, whereas the count of participants experiencing events was divided for dichotomous outcomes.

Considering the potential influence of false-positive outcomes resulting from multiple testing and limited data on the study’s findings, an assessment of the robustness of the cumulative evidence on the primary outcome (i.e. time to flatus) was performed using trial sequential analysis (TSA) (TSA viewer version 0.9.5.10 Beta). The interplay between the TSA boundary and cumulative Z-curve was examined after the computation of trial sequential monitoring and required information size thresholds. The cumulative *Z*-curve crossing the TSA boundary indicates a substantiated level of evidence in support of the anticipated intervention effect, thereby obviating the need for further studies. Conversely, the failure of the cumulative *Z*-curve and TSA boundary to intersect signifies insufficient evidence to draw a reliable conclusion.

The statistical analyses for this meta-analysis were conducted with the oversight of an experienced statistician to ensure methodological rigor and accuracy. The statistician reviewed the data extraction, the choice of models, the interpretation of test statistics, and the substantiation of the conclusions drawn from the data.

## Results

### Study selection and characteristics of studies

**Figure 1:**
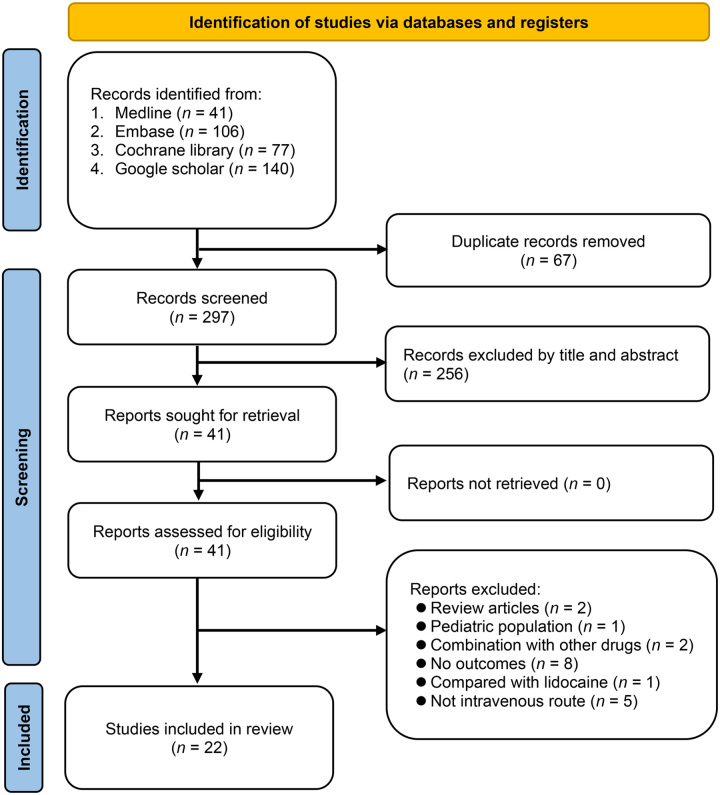
An overview of the selection process for studies.

The initial database search yielded 364 records, of which 67 duplicate records were identified and removed. Following the title and abstract evaluation of the remaining 297 records, 41 were selected for the full-text review stage. Among these, a comprehensive assessment excluded 19 records for various reasons, as illustrated in Figure 1. Consequently, our meta-analysis encompassed 22 RCTs^20–41^, all of which were published between 2008 and 2023. An overview of the study selection process is shown in Figure 1.

The key characteristics of the RCTs included in our meta-analysis are summarized in Table 1. Twenty of the 22 RCTs specifically enrolled patients aged less than 70 years. In two distinct RCTs, the patients had a median or mean age of 70 years. Regarding sex distribution, 15 of the included studies deliberately incorporated a mixed-sex population, with male participation ranging from 23.8 to 81%. In contrast, a subset of seven RCTs exclusively enrolled female patients^25,29,32,37,38,40,41^. A wide range of participant counts was observed in all RCTs, ranging from 34 to 675. Consequently, a total of 2566 patients were collectively analyzed. Fifteen of the 22 studies employed the laparoscopic technique for both upper and lower abdominal surgeries^20–25,27,31,32,34,36–38,40,41^. In contrast, the remaining six studies included patients undergoing a diverse range of procedures, including abdominal and nonabdominal surgeries, and employed nonlaparoscopic methodologies, including spinal fusion or open colectomy^26,28–30,35,39^. One study examined patients who underwent laparoscopic or open colorectal surgery^33^. Of the 22 RCTs, 17 involved intraoperative Dex administration^20–27,30–32,34–36,38,40,41^, three allocated this intervention postoperatively^33,37,39^, and two adopted a dual approach^28,29^, encompassing both intraoperative and postoperative phases. Of the 17 included studies involving intraoperative Dex, 0.2–0.6 µg/kg/h was the most frequently used infusion rate and was observed in 15 studies^20–25,27,31,32,34–36,38,40,41^. Geographically, most of these RCTs (21/22 RCTs) were conducted in Asia. Specifically, China, Korea, India, and Taiwan contributed 17, 2, 1, and 1 studies, respectively. Only one study has been conducted in the United States.

**Table 1:** Characteristics of studies (*n*=22).

Studies	Age (years); D vs. C	Male (%)	BMI (kg/m^2^); D vs. C	N	Type of surgery	Dex dosage (loading; infusion)	Country
Chen *et al*. 2016^20^	57 vs. 60	48.3	60 vs. 59^a^	60	Colorectal surgery^b^	1 μg/kg; 0.3 μg/kg/h	China
Cho *et al*. 2015^21^	55 vs. 55	54.4	23 vs. 23	90	Gastrectomy^b^	0.5 μg/kg; 0.4 μg/kg/h	Korea
Guo *et al*. 2022^22^	69	51.1	64^a^	90	Gastrectomy^b^	1.0 μg/kg; 0.5 μg/kg	China
Huai *et al*. 2022^23^	62 vs. 64	31.3	68^a^	80	Colon surgery^b^	0.5 μg/kg; 0.4 μg/kg/h	China
Huang *et al*. 2021^24^	D1: 52, D2: 52; C: 53	48.3	D1: 26; D2: 26; C: 26	120	Nephrectomy^b^	D1: 0.2 μg/kg/h D2: 0.4 μg/kg/h	China
Koo *et al*. 2023^25^	42 vs. 44	0	58 vs. 59^a^	96	Gynecological Laparoscopy^b^	0.7 μg/kg; 0.5 μg/kg/h	Korea
Li *et al*. 2019^26^	59 vs. 61	40.9	25 vs. 26	66	Spinal Fusion	0.5 μg/kg; 0.1 μg/kg/h	China
Lu *et al*. 2021^27^	70 vs. 70	65.9	22 vs. 22	675	Abdominal surgery^b^	0.5 μg/kg; 0.2 μg/kg/h	China
Mao *et al*. 2020^28^	65 vs. 63	81	20 vs 21	58	Thoracotomy	0.5 μg/kg; 0.2–0.4 μg/kg/h^c^	China
Nie *et al*. 2018^29^	30 vs. 31	0	27 vs. 27	205	Cesarean delivery	0.5 μg/kg; Postoperative PCA infusion: 3 μg/h^d^	China
Ou *et al*. 2022^30^	55 vs. 55	62.8	NA	102	Open colectomy	1 μg/kg; 1.5 μg/kg/h	China
Qin *et al*. 2023^31^	70 vs. 70	70.3	23 vs. 23	64	Colorectal surgery^b^	0.4 μg/kg; 0.5 μg/kg/h	China
Sivaji *et al*. 2021^32^	46 vs. 47	0	24 vs. 24	48	Hysterectomy^b^	1 μg/kg; 0.6 μg/kg/h	India
Sui *et al*. 2022^33^	D1:59; D2: 60; C: 60	64.7	D1:67; D2: 70; C: 66^a^	210	Colorectal surgery: Laparoscopy (52%)	200 μg or 400 μg was mixed with PCA^e^	China
Sun *et al*. 2021^34^	60 vs. 59	64.3	23 vs. 24	56	Colorectal surgery^b^	1 μg/kg; 0.5 μg/kg/h	China
Tseng *et al*. 2021^35^	31 vs. 35	44.1	24 vs. 22	34	Open hepatectomy	0.4 μg/kg/h	Taiwan
Tufanogullari *et al*. 2008^36^	D1: 47; D2:48 D3:40; C: 43	23.8	D1:127; D2: 138; D3: 151; C: 127^a^	80	Bariatric surgery^b^	D1 0.2 μg/kg/h D2 0.4 μg/kg/h D3 0.8 μg/kg/h	USA
Wang *et al*. 2016^37^	45 vs. 50	0	24 vs. 26	36	Gynecological surgery^b^	PCA infusion: 0.25 μg/kg/h^f^	China
Wu *et al*. 2022^38^	47 vs. 47	0	27 vs. 26	106	Hysteromyomectomy^b^	0.5 μg/kg; 0.2 μg/kg/h	China
Xin *et al*. 2017^39^	51 vs. 55	54.8	63 vs. 63^a^	93	laparotomy surgery	PCA infusion: 0.04 μg/kg/h^f^	China
Xu *et al*. 2017^41^	48 vs. 47	0	24 vs. 25	120	Abdominal hysterectomy^b^	0.5 μg/kg; 0.4 μg/kg/h	China
Xu *et al*. 2021^40^	48 vs. 47	0	24 vs. 25	80	Hysterectomy^b^	0.5 μg/kg; 0.4 μg/kg/h	China

a Weight (kg).

b Laparoscopic approach.

c Postoperative 5 days.

d One day.

e Three days.

f Two days.

C, control group; D, dexmedetomidine group.

### Risk of bias

**Figure 2:**
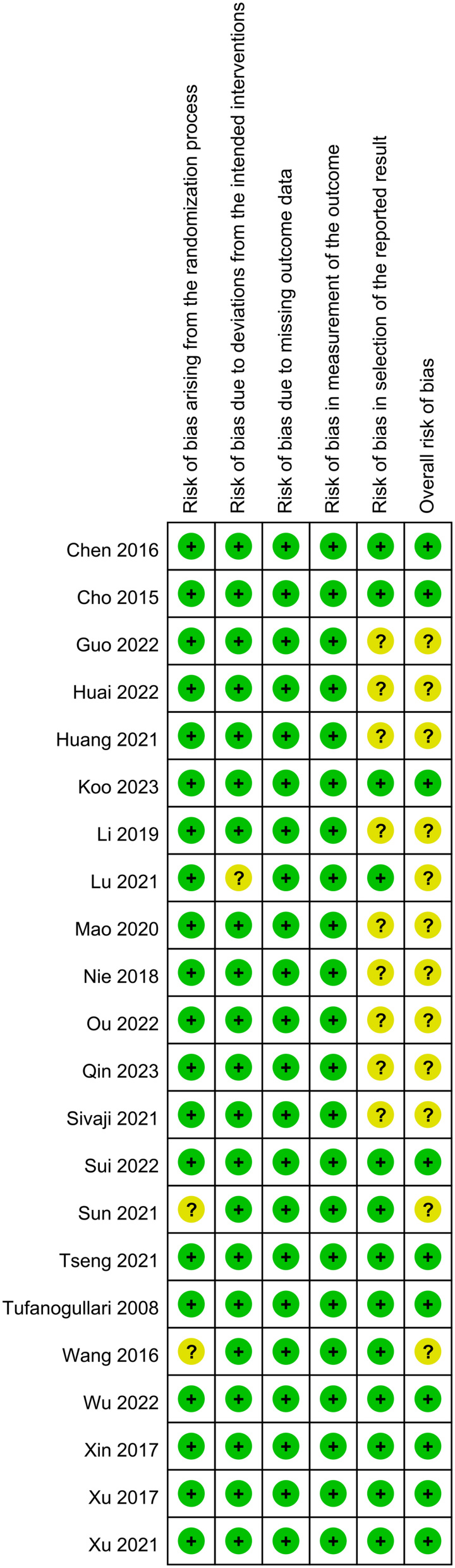
The risks of bias of individual studies.

The randomization process demonstrated adequacy with a low risk of bias in the majority of studies, whereas only two studies raised concerns (Figure 2). Deviations from interventions and missing outcome data were effectively managed, with a low risk of bias across all studies, and the methodology for outcome measurement was suitable in every study. However, a selective reporting bias was noted in 12 studies. In general, the overall risk of bias was low in most studies (15/22 RCTs) and slightly concerning in seven studies. Despite a few selective outcome reporting issues, the included RCTs exhibited commendable methodology and appropriate study conduct, yielding a low risk of bias across most parameters.

### Outcomes

#### Primary outcome

**Figure 3:**
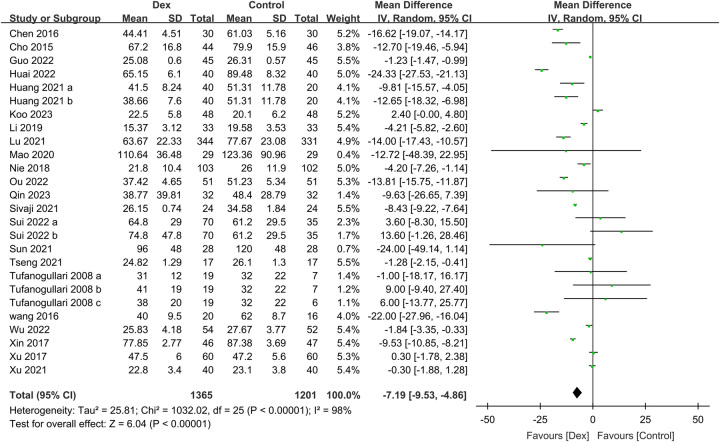
Forest plot showing the association of intravenous dexmedetomidine (Dex) use with the time to first flatus. IV, inverse variance.

Twenty-two studies contributed data on the correlation between intravenous Dex and time to flatus, consisting of a Dex group of 1365 patients and a control group of 1201 patients. This meta-analysis indicated a significantly shorter time to flatus with Dex than without Dex (MD: −7.19 h, 95% CI: −9.53 to −4.86, *P*<0.00001, *I* ^2^=98%) (Fig. 3). This finding was robust in sensitivity analyses that employed the leave-one-out method. Furthermore, a minimal likelihood of publication bias was observed for the primary outcome (Supplementary Figure 1, Supplemental Digital Content 5, http://links.lww.com/JS9/B568).

**Figure 4:**
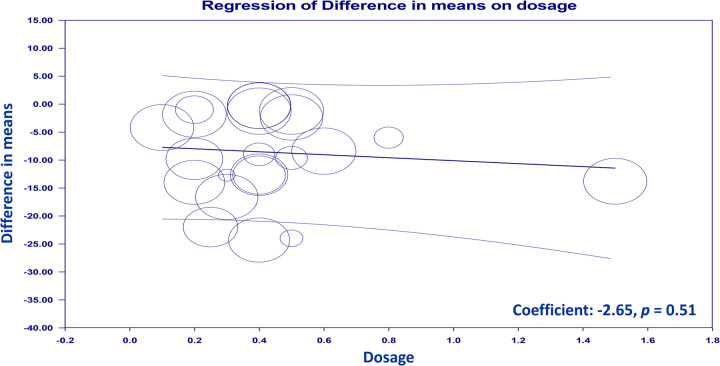
Meta-regression indicating a lack of correlation between infusion dosage and the beneficial effects of intravenous dexmedetomidine (Dex) on time to flatus (coefficient: −1.26, *P*=0.069).

**Figure 5:**
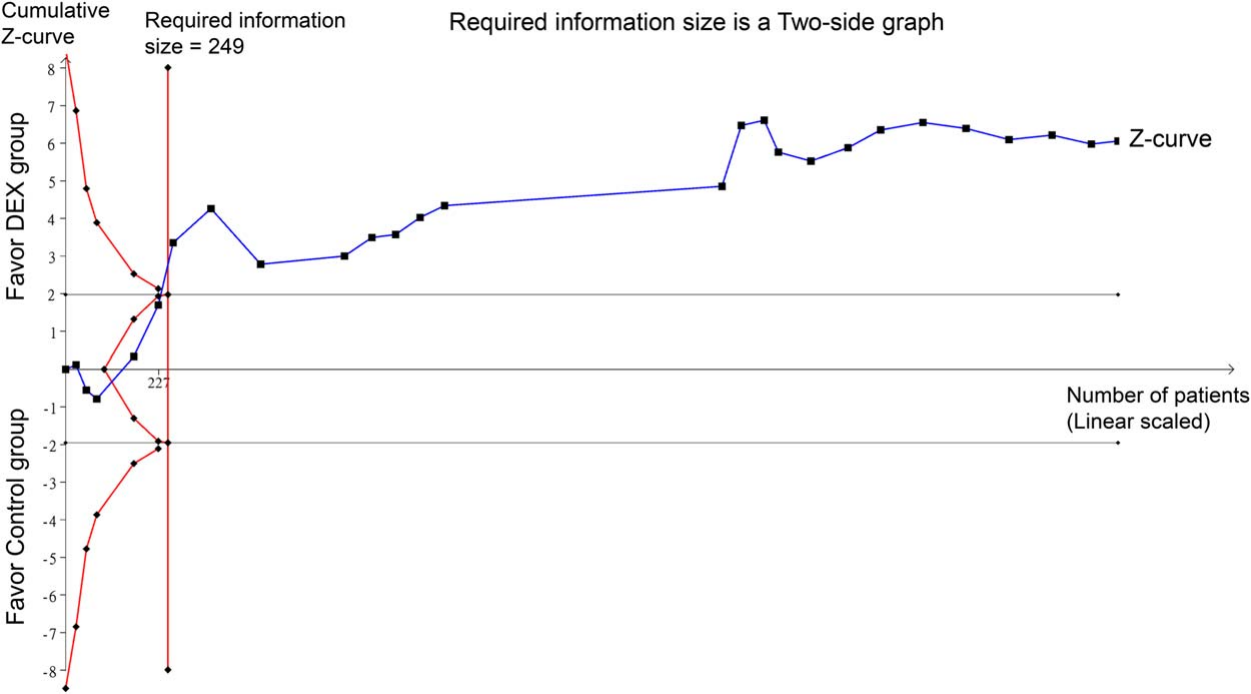
Trial sequential analysis supporting the substantial evidence regarding intravenous dexmedetomidine’s impact on time to flatus.

Subgroup analyses were conducted to explore the influence of surgical approach type and patient sex on the efficacy of intravenous Dex in time to first flatus. The detailed results of these analyses are presented in Supplementary Figures 2 (Supplemental Digital Content 5, http://links.lww.com/JS9/B568) to 4. We found that intravenous Dex significantly reduced the time to first flatus in patients across various surgical categories, including those undergoing laparoscopic and nonlaparoscopic procedures (Supplementary Figures 2, Supplemental Digital Content 5, http://links.lww.com/JS9/B568), as well as abdominal and nonabdominal surgeries (Supplementary Figures 3, Supplemental Digital Content 5, http://links.lww.com/JS9/B568). Furthermore, the beneficial effects of Dex on hastening bowel motility were consistent in both mixed-sex and female-only subgroups (Supplementary Figures 4, Supplemental Digital Content 5, http://links.lww.com/JS9/B568). These findings suggest that the impact of intravenous Dex on gastrointestinal function recovery is robust across different surgical contexts and patient sexes. Conversely, when considering country-based subgroup analysis, it became evident that the pooled data from regions beyond China did not exhibit the same advantageous trend in favor of intravenous Dex (Supplementary Figure 5, Supplemental Digital Content 5, http://links.lww.com/JS9/B568). Meta-regression analysis results indicated that the infusion dosage of intravenous Dex did not exert an advantageous effect on the time to flatus (coefficient: −1.26, *P*=0.069) (Fig. 4). TSA showed a noteworthy crossing of the *Z*-curve with the RIS, highlighting robust evidence supporting the favorable impact of intravenous Dex on the time to flatus (Fig. 5).

#### Secondary outcomes

**Figure 6:**
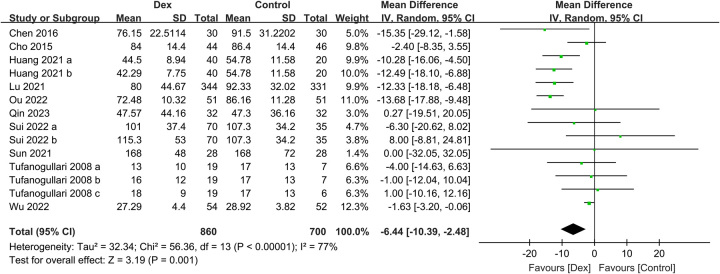
Forest plot showing the association of intravenous dexmedetomidine (Dex) use with the time to oral feeding. IV, inverse variance.

**Figure 7:**
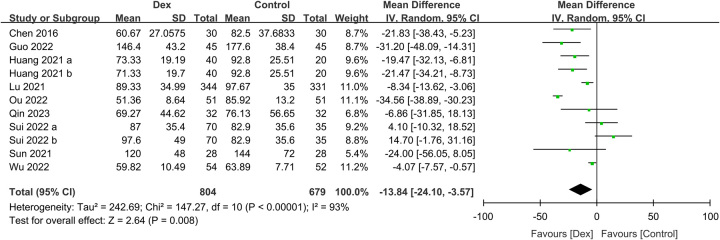
Forest plot showing the association of intravenous dexmedetomidine (Dex) use with the time to defecation. IV, inverse variance.

**Figure 8:**
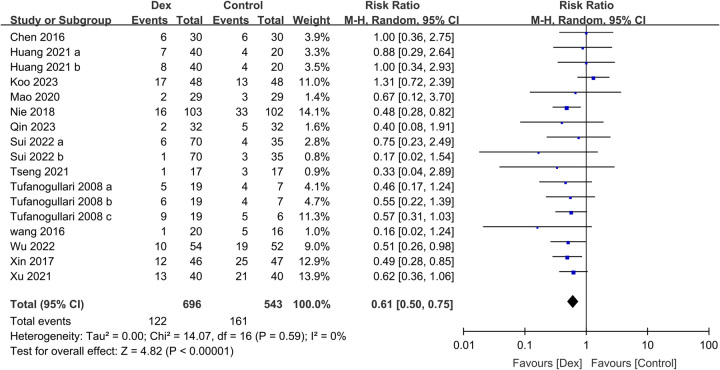
Forest plot showing the association of intravenous dexmedetomidine (Dex) use with the risk of postoperative nausea and vomiting (PONV).

**Figure 9:**
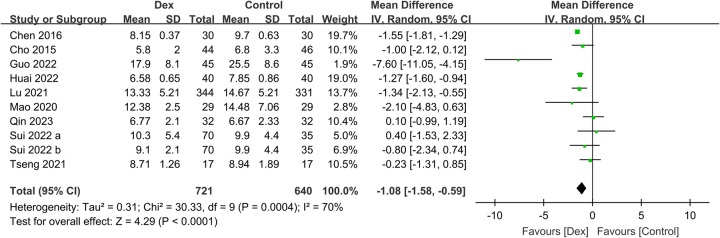
Forest plot showing the association of intravenous dexmedetomidine (Dex) use with the length of hospital stay (LOS). IV: inverse variance.

Participants who received intravenous Dex had a faster time to oral feeding than those who did not receive it (MD: −6.44 h, 95% CI: −10.39 to −2.48, *P*=0.001, *I* ^2^=77%, sensitivity analysis: consistent, 1560 participants) (Fig. 6). Similarly, those who received intravenous Dex had a shorter time to defecation than those who did not receive it (MD: −13.84 h, 95% CI: −24.1 to −3.57, *P*=0.008, *I* ^2^=93%, sensitivity analysis: consistent, 1483 participants) (Fig. 7). There were no differences in pain severity at 12–24 h (MD: −0.1 point, 95% CI: −0.25–0.06, *P*=0.22, *I* ^2^=49%, sensitivity analysis: consistent, 1043 participants) (Supplemental Figure 6, Supplemental Digital Content 5, http://links.lww.com/JS9/B568), the risks of hypotension (RR:1.0, 95% CI: 0.56–1.79, *P*=0.99, *I* ^2^=40%, sensitivity analysis: consistent, 360 participants) (Supplemental Figure 7, Supplemental Digital Content 5, http://links.lww.com/JS9/B568), and bradycardia (RR: 1.28, 95% CI: 0.6–2.74, *P*=0.52, *I* ^2^=62%, sensitivity analysis: consistent, 711 participants) (Supplemental Figure 8, Supplemental Digital Content 5, http://links.lww.com/JS9/B568). The risk of PONV were lower in the Dex group than in the control group (RR: 0.61, 95% CI: 0.5–0.75, *P*<0.00001, *I* ^2^=0%, sensitivity analysis: consistent, 1239 participants) (Fig. 8). Moreover, the Dex group had a shorter LOS than the control group (MD: −1.08 days, 95% CI: −1.58 to −0.59, *P*<0.0001, *I* ^2^=70%, sensitivity analysis: consistent, 1361 participants) (Fig. 9).

The funnel plot demonstrated low risks of bias on the time to oral feeding (Supplemental Figure 9, Supplemental Digital Content 5, http://links.lww.com/JS9/B568), time to defecation (Supplemental Figure 10, Supplemental Digital Content 5, http://links.lww.com/JS9/B568), pain severity at 12–24 h (Supplemental Figure 11, Supplemental Digital Content 5, http://links.lww.com/JS9/B568), risk of PONV (Supplemental Figure 12, Supplemental Digital Content 5, http://links.lww.com/JS9/B568), and LOS (Supplemental Figure 13, Supplemental Digital Content 5, http://links.lww.com/JS9/B568).

## Discussion

This meta-analysis showed that Dex is associated with quicker times to flatus, oral feeding, and defecation, reduced PONV risk, and shorter hospital stays, with no added risk of hemodynamic instability or increased postoperative pain score. Subgroup analyses supported the positive impact of Dex on the time to flatus across different surgical approaches and sexes. However, this advantageous trend has not been observed in studies conducted in regions outside China. Meta-regression analysis showed that the Dex dosage had no influence on the time to flatus. TSA provided strong evidence supporting the favorable impact of intravenous Dex on time to flatus.

The mechanisms of POI vary, and include autonomic regulation, inflammatory response, gastrointestinal hormones, and postoperative use of opioid drugs. Damage to the gut during surgery can compromise the intestinal barrier, activate the sympathetic and parasympathetic nervous systems, and increase the release of inflammatory factors, each of which may play a role in POI development^3,24,42^. Previous studies consistently showed that by reducing sympathetic ganglion fiber neurotransmitter release, Dex safeguards organ function^43^. This action curtails inflammatory factor production, lowers oxidative stress, and mitigates reperfusion injury in both human and animal studies^26,44,45^. Additionally, Dex dose-dependently inhibits the production of neutrophil chemokines, CXCL1 and CXCL2, in a carrageenan-induced mouse air pouch inflammation model^46^. Moreover, intraoperative Dex administration has been associated with improved markers of intestinal injury and permeability, including D-lactate, serum diamine oxidase (DAO) activity, and intestinal fatty-acid binding protein (I-FABP) levels^22,34,47^. In Kuru’s study, Dex exhibited substantial adhesion-preventive effects in rats, which were attributed to its antioxidant and anti-inflammatory properties^48^. Additionally, the postoperative levels of serum gastrin and plasma motilin, which are beneficial for gastrointestinal function, are higher in the Dex-administered groups, whereas plasma cholecystokinin levels, which may hinder gastrointestinal motility, are lower than those in the control groups^30^.

Flatus recovery time is considered a gastrointestinal function recovery indicator, with a recent review frequently identifying it as the primary outcome measure for assessing postoperative bowel function recovery^19^. Moreover, prolonged flatus recovery time is associated with an increased risk of POI, which can further impact a patient’s timing of initiating oral intake. In this meta-analysis, we discovered that Dex significantly reduced the time to flatus, with an MD of −7.19 h (*P*<0.00001). This finding aligns well with that of a previous meta-analysis, which noted a 5.61 h decrease^16^. The comparatively quicker time to flatus in our meta-analysis may have resulted from our specific inclusion criteria, encompassing only studies that employed standard care, placebos (e.g. normal saline), or opioids as control groups. In contrast, a recent meta-analysis also incorporated studies that utilized lidocaine as a comparator^16^. Considering that perioperative lidocaine infusion has a favorable influence on gastrointestinal function recovery^49,50^, the advantageous effects of Dex could be obscured if more discerning selection of the control group is not performed. Nonetheless, our meta-analysis showed a substantial heterogeneity (*I* ^2^=98%). Subgroup analyses focusing on surgical approach type, sex, and country did not appear to mitigate this heterogeneity. We propose that other unidentified variables, including disparities in medical care, may contribute to the observed heterogeneity. Despite these considerations, TSA validates the robustness of the evidence, thus validating Dex administration in patients with POI poses a concern.

Our subgroup analyses showed variations in the effects of Dex on the time to flatus, depending on sex and geographical location. Although Dex consistently reduced the time to flatus across both mixed-sex and female-only groups, the reduction was somewhat less pronounced in the female-only subgroup (i.e. 4.33 h) than in the mixed-sex subgroup (8.59 h) (Supplementary Figure 4, Supplemental Digital Content 5, http://links.lww.com/JS9/B568). Previous studies identified male sex as a risk factor for POI^51,52^. Consequently, the positive effects of Dex may be more evident in males than females. Additionally, subgroup analysis indicated that Dex reduced the time to flatus by −8.5 h in Chinese studies, whereas no significant difference was observed in studies from other countries. Such variations may stem from disparities in surgical methods, anesthetic practices, patient characteristics, and perioperative care between China and other countries. Further studies are needed to explore the influence of geographical location on the beneficial effects of Dex. Regarding surgical approaches, our subgroup analysis supported Dex administration in patients undergoing laparoscopic or nonlaparoscopic surgeries (Supplementary Figure 2, Supplemental Digital Content 5, http://links.lww.com/JS9/B568), despite laparoscopic approach being less invasive^24,53^. Moreover, the role of Dex in accelerating gastrointestinal function is not confined to abdominal surgeries, which directly involve bowel manipulation; it also aids in the recovery of gastrointestinal motility following nonabdominal surgeries (Supplementary Figure 3, Supplemental Digital Content 5, http://links.lww.com/JS9/B568). This is particularly relevant in instances where ileus may develop because of factors such as postoperative immobility and the use of perioperative opioids. Our findings reinforce the versatility of Dex as an adjunct in perioperative care with the potential to facilitate better postoperative outcomes across a diverse range of surgical contexts.

Beyond the time to flatus, our meta-analysis consistently demonstrated that Dex administration leads to earlier initiation of oral intake and quicker defecation, thereby reinforcing its positive impact on gastrointestinal function recovery. Furthermore, we noted that patients who received Dex had shorter LOS (−1.08 days) than those who did not receive it. This finding indirectly supports the idea that medical resources can be optimized by reducing POI risk. Conversely, a recent meta-analysis, while acknowledging the positive correlation between Dex administration and time to flatus, did not find it beneficial in reducing the time to oral intake or LOSs^16^. The discrepancies in findings may result from the inclusion of a limited number of studies in that meta-analysis^16^, potentially underestimating the beneficial effects. Furthermore, the inclusion of studies using lidocaine as a comparator in that meta-analysis may obscure the potential benefits of Dex^16^.

The low risk of PONV in the current meta-analysis may be attributed to the antiemetic effects of Dex through its intrinsic α2 agonist antiemetic properties or the potential for indirect opioid-sparing mechanisms^54,55^. Furthermore, a shorter time to flatus may be associated with a reduced risk of abdominal distention, which further reduces the risk of PONV.

Common adverse effects with the use of Dex include bradycardia, hypotension, and a dry mouth^56,57^. Less commonly, it can cause hypertension, nausea, and fever. Owing to its effects on the cardiovascular system, careful consideration is required in patients with severe ventricular dysfunction, conduction disturbances, and chronic hypertension, as it may exacerbate these conditions^56,57^. Contraindications for Dex include known hypersensitivity to the drug, and caution is advised in cases where the suppression of the central nervous system activity could be detrimental, such as in patients with advanced heart block or severe hypovolemia^56,57^.

The optimal dosage range for Dex that offers pain relief while preventing adverse effects on the gastrointestinal system and cardiovascular function remains to be elucidated. Adjunctive Dex administration at a relatively slow rate of 0.4 µg/kg/h during abdominal surgery has been proven effective for opioid-sparing and mitigating acute postoperative pain^35,58^. However, higher infusion rates such as 1.0 µg/kg/h may pose risks of hypotension or bradycardia^59,60^. Additionally, a previous study reported that intravenous administration of Dex at 1 µg/kg over 20 min followed by a continuous 0.7 µg/kg/h infusion for ~3 h inhibited gastric emptying in healthy male participants^61^. These findings suggest that higher dosages have unfavorable gastrointestinal and cardiovascular effects. Among the 17 included studies on intraoperative Dex, 15 predominantly used an infusion rate of 0.2–0.6 µg/kg/h. Our meta-regression analysis indicated no dose-dependent effect on the time to flatus, suggesting the clinical viability of this dosage range. Considering the absence of significant differences in the risks of hypotension or bradycardia in the current meta-analysis, Dex dosages of 0.2–0.6 µg/kg/h appear to be clinically advantageous and safe.

Our study has some limitations. First, significant heterogeneity was noted in the use of Dex, particularly in the postoperative settings. This can be attributed to variations in dosages and administration methods, which could have affected the consistency of the results. Second, the majority of the included studies were conducted in Asia, with 17 of the 22 originating from China. Only one study originated from a Western country, which may limit the generalizability of the findings to Western populations. Lastly, different surgeries had varying sex and age distributions among patients, and the original RCTs did not perform sex-specific and age-specific analyses. Therefore, to determine how to maximize the gastrointestinal protective effects of Dex with minimal side effects in different surgical categories and among different sexes and age groups, considering patient-specific factors, further studies and analyses are needed.

## Conclusion

Our meta-analysis suggested that Dex administration was significantly associated with reduced time to flatus, expedited initiation of oral feeding, quicker time to defecation, decreased risk of PONV, and shortened hospital stay, without altering the risk of hemodynamic instability and postoperative pain score. Due to its ability to promote gastrointestinal recovery, Dex may serve as a supplemental analgesic agent during the perioperative period for patients undergoing abdominal and nonabdominal surgeries, especially when reducing the risk of POI is a priority. Cost-effectiveness analysis and investigation of the non-Asian population could further elucidate the role of perioperative Dex in improving postoperative outcomes.

## Ethical approval

Not applicable.

## Consent

Not applicable.

## Sources of funding

Not applicable.

## Author contribution

Y.-C.L. and K.-C.H.: conceptualization, methodology, and software; W.-T.W. and J.-Y.W.: data curation; K.-C.H. and I.-W.C.: writing – original draft preparation; J.-Y.C. and C.-M.L.: visualization and investigation; I.-W.C.: supervision; Y.-J.C. and I.-W.C.: software and validation; K.-C.H. and I.-W.C.: writing– reviewing and editing.

## Conflicts of interest disclosure

The authors declare that they have no financial conflict of interest with regard to the content of this report.

## Research registration unique identifying number (UIN)

Prospero registration number: CRD42023455540.

## Guarantor

Kuo-Chuan Hung and I-Wen Chen.

## Data availability statement

The datasets used and/or analyzed in the current study are available from the corresponding author upon reasonable request.

## Provenance and peer review

Not commissioned, externally peer-reviewed.

## Supplementary Material

SUPPLEMENTARY MATERIAL

## Supplementary Material

**Figure:**