# Supplementary material for: Impact of intravenous dexmedetomidine on postoperative gastrointestinal function recovery: an updated meta-analysis
Source: Int J Surg. 2023 Dec 12;110(3):1744–54. doi: 10.1097/JS9.0000000000000988 (PMC10942148; doi:10.1097/JS9.0000000000000988)
Supplement: SUPPLEMENTARY MATERIAL [file js9-110-1744-s004.docx]

**Supplemental Table 1.** Search strategy for Medline

| 1 | ("Dexmedetomidine" or "Precedex").mp. |
| --- | --- |
| 2 | exp "Dexmedetomidine"/ |
| 3 | ("postoperative ileus" or "postoperative bowel dysfunction" or "postoperative gastrointestinal dysfunction" or "paralytic ileus" or "Surgical ileus" or "Gastrointestinal recovery" or "Gastrointestinal motility" or "Flatus" or "Feces" or "Defecation" or "Bowel function" or "bowel recovery").mp. |
| 4 | exp "ileus"/ |
| 5 | (1 or 2) and (3 or 4) |
| 6 | 5 and (((randomized controlled trial or controlled clinical trial).pt. or randomi*ed.ab. or placebo.ab. or drug therapy.fs. or randomly.ab. or trial.ab. or groups.ab.) not (exp animals/ not humans.sh.)) |
