# Supplementary material for: Impact of intravenous dexmedetomidine on postoperative gastrointestinal function recovery: an updated meta-analysis
Source: Int J Surg. 2023 Dec 12;110(3):1744–54. doi: 10.1097/JS9.0000000000000988 (PMC10942148; doi:10.1097/JS9.0000000000000988)

**Supplemental Figure 1.** Funnel plot indicating a low risk of bias on the association between intravenous dexmedetomidine use and time to flatus.


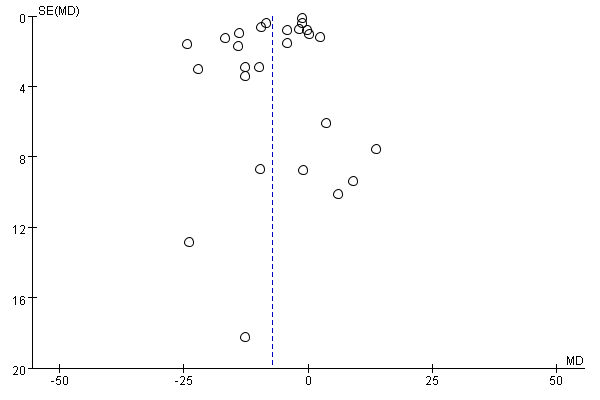


**Supplemental Figure 2.** Subgroup analysis based on laparoscopic versus non–laparoscopic surgery.


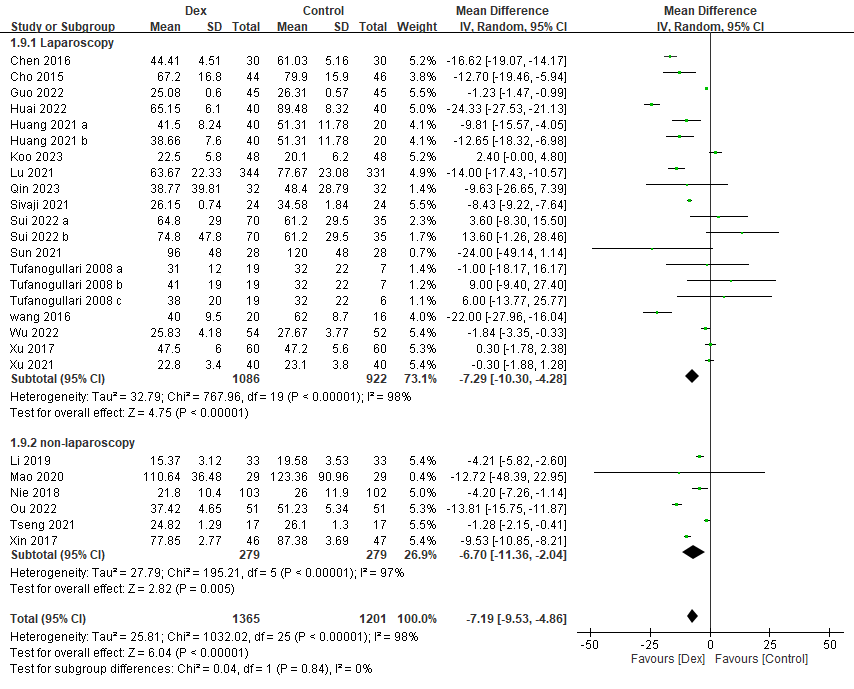


**Supplemental Figure 3.** Subgroup analysis based on abdominal vs. non-abdominal surgery

**
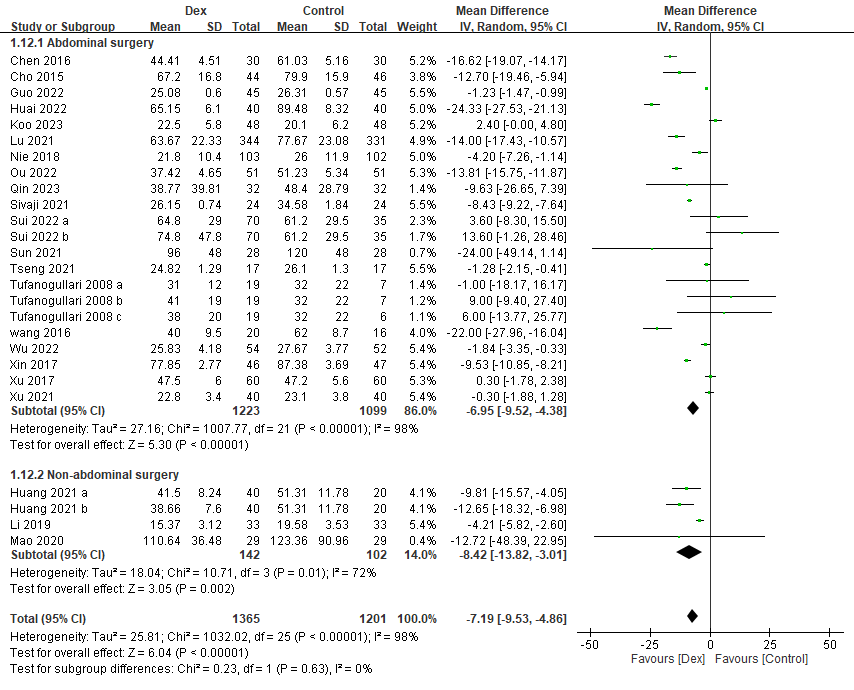
**

**Supplemental Figure 4:** Subgroup analysis was performed based on gender (i.e., mixed gender vs. female).

**
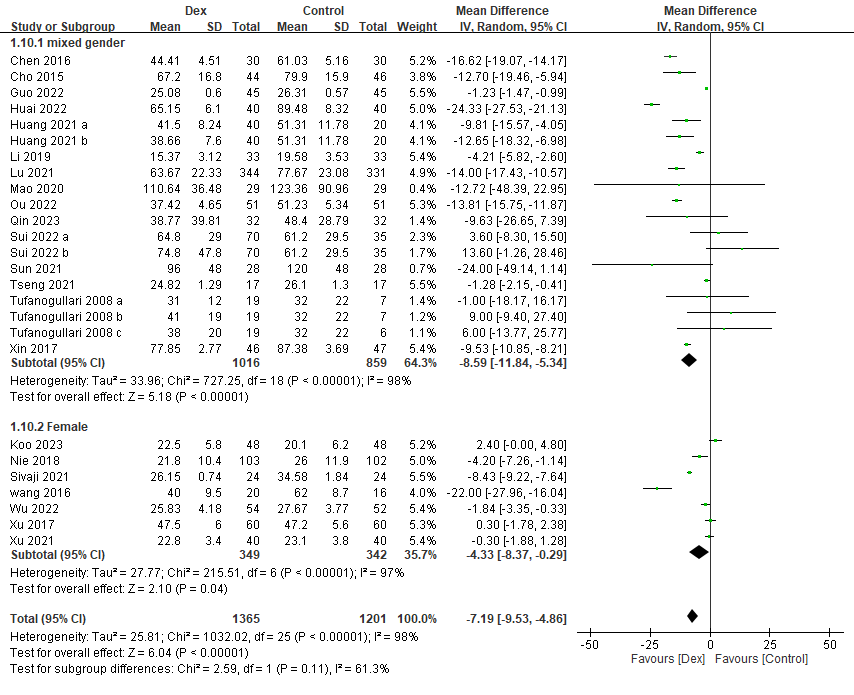
**

**Supplemental Figure 5:** Subgroup analysis was performed based on country (i.e., China vs. non-China).


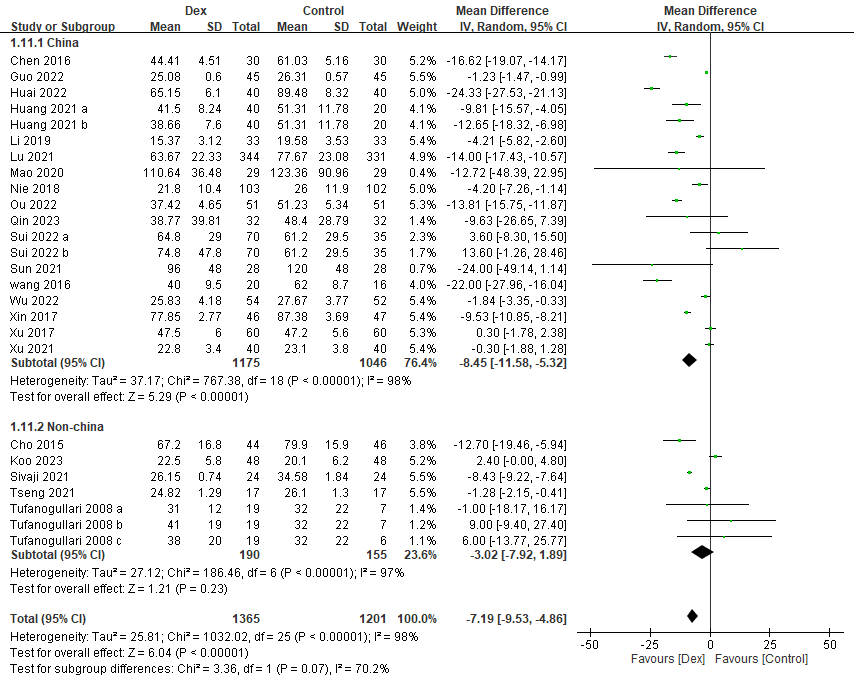


**Supplemental Figure 6:** Forest plot showing the association of intravenous dexmedetomidine (Dex) use with the pain severity at 12–24 h. IV: inverse variance; CI: confidence interval; SD: standard deviation.

**
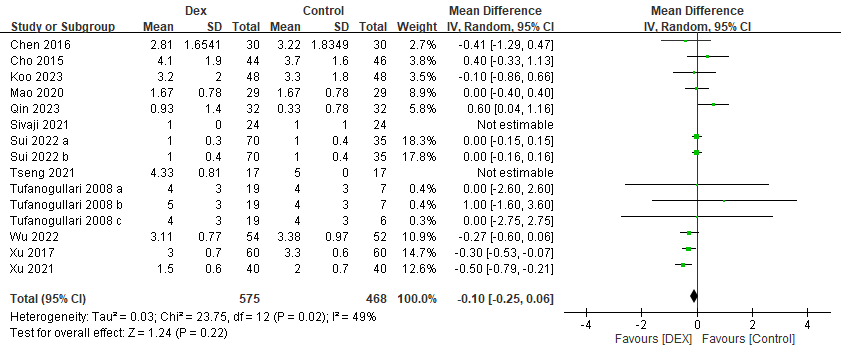
**

**Supplemental Figure 7:** Forest plot showing the association of intravenous dexmedetomidine (Dex) use with the risk of hypotension. CI: confidence interval.

**
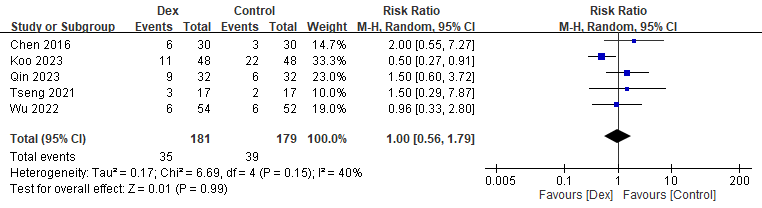
**

**Supplemental Figure 8:** Forest plot showing the association of intravenous dexmedetomidine (Dex) use with the risk of bradycardia. CI: confidence interval.

**
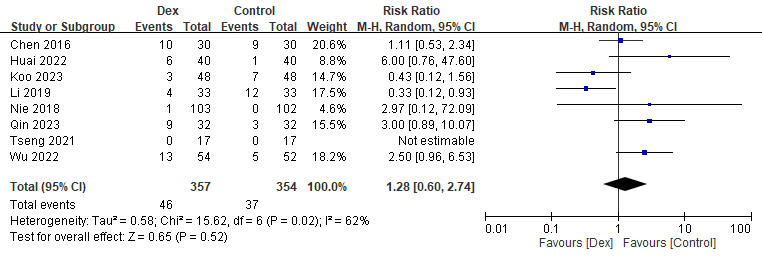
**

**Supplemental Figure 9.** Funnel plot showing a low risk of bias between intravenous dexmedetomidine and time to oral feeding.


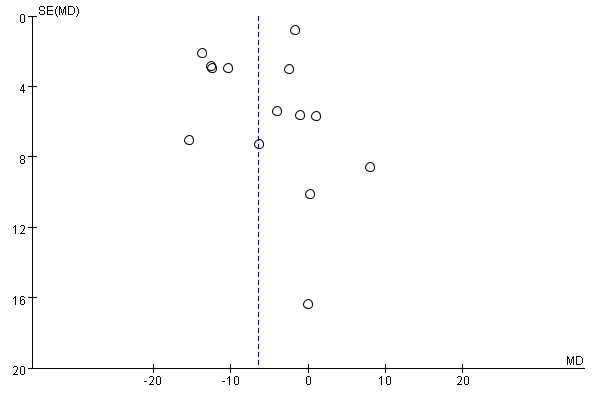


**Supplemental Figure 10.** Funnel plot showing a low risk of bias between intravenous dexmedetomidine and time to defecation.


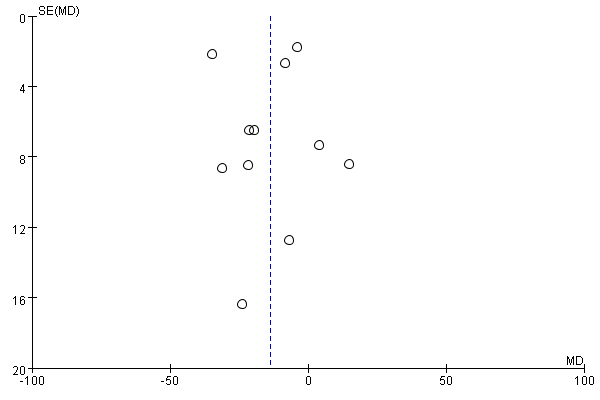


**Supplemental Figure 11.** Funnel plot showing a low risk of bias between intravenous dexmedetomidine and pain severity at 12-24 hours


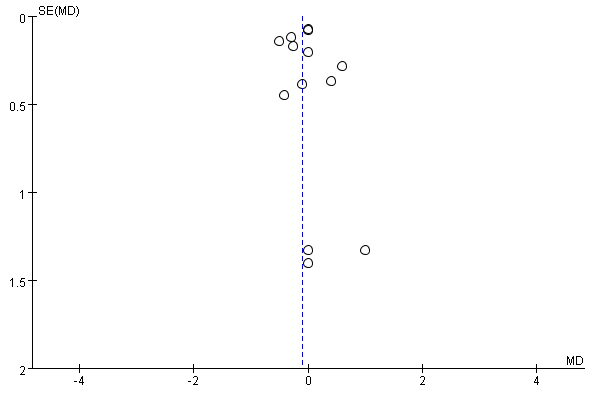


**Supplemental Figure 12.** Funnel plot showing a low risk of bias between intravenous dexmedetomidine and risk of postoperative nausea and vomiting


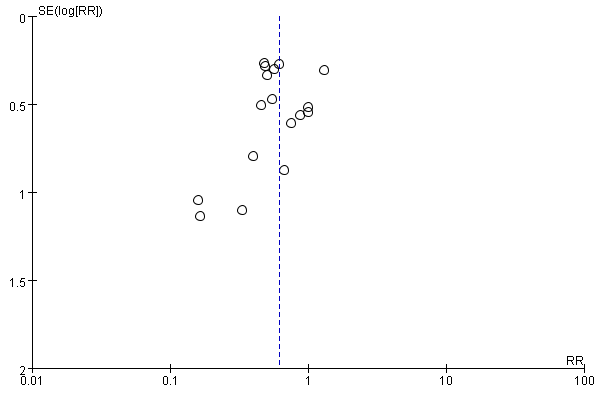


**Supplemental Figure 13.** Funnel plot showing a low risk of bias between intravenous dexmedetomidine and length of hospital stay


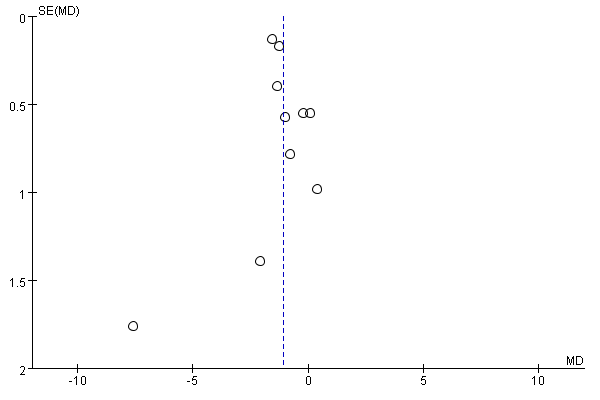

Supplement: SUPPLEMENTARY MATERIAL [file js9-110-1744-s005.docx]
